# Microsatellites for the Neotropical Ant, *Odontomachus chelifer* (Hymenoptera: Formicidae)

**DOI:** 10.1093/jisesa/ieaa117

**Published:** 2020-10-24

**Authors:** Alessandra S M Lemos, Marianne Azevedo-Silva, Salatiel Gonçalves-Neto, Anete P Souza, Paulo S Oliveira

**Affiliations:** 1 Graduação em Ciências Biológicas, Departamento de Biologia Animal, Universidade Estadual de Campinas, Campinas, SP, Brazil; 2 Programa de Pós-Graduação em Ecologia, Departamento de Biologia Animal, Universidade Estadual de Campinas, Campinas, SP, Brazil; 3 Departamento de Biologia Vegetal, Centro de Biologia Molecular e Engenharia Genética, Universidade Estadual de Campinas, Campinas, SP, Brazil; 4 Departamento de Biologia Animal, Universidade Estadual de Campinas, Campinas, SP, Brazil

**Keywords:** molecular marker, simple sequence repeat, Ponerinae, Hymenoptera, social insect

## Abstract

*Odontomachus chelifer* (Latreille) (Ponerinae) is a ground-dwelling, predominantly carnivorous ant whose colonies may contain multiple egg-laying queens and are potentially susceptible to border effects in the Brazilian savanna known as Cerrado. The ecology and natural history of *O. chelifer* is well studied, but very little is known about the genetic diversity of *O. chelifer* colonies. In this study, we developed microsatellite markers for the study of genetic variation in *O. chelifer*. We created a microsatellite-enriched library that resulted in the development and characterization of 22 markers, of which 18 were found to be polymorphic in the population studied. The mean expected heterozygosity was 0.59, whereas the mean rarified allelic richness was determined as 4.27 alleles per locus. The polymorphism level detected was similar to genetic diversity estimates found in other poneromorph ant species. The microsatellites developed here are likely to be useful for the investigation of colony structure, functional polygyny, breeding system, and population genetics in *O. chelifer*. Moreover, the description of *O. chelifer*’s genetic diversity is crucial for its conservation and maintenance of its ecological role in the Cerrado savanna.

*Odontomachus chelifer* (Latreille) (Formicidae: Ponerinae) (Fig. 1) is a ground-dwelling, nocturnal ant that is widely distributed in the Neotropical region, occurring in forest and savanna habitats from Mexico to Argentina (Kempf 1972). The species is predominantly carnivorous and feeds on litter-dwelling arthropods, mostly termites (Raimundo et al. 2009). Additionally, members of this species frequently harvest lipid- and protein-rich fleshy fruits and seeds, which are fed to larvae in the nest (Passos and Oliveira 2002, 2004; Bottcher and Oliveira 2014). *Odontomachus chelifer* is facultatively polygynous (i.e., more than one egg-laying queen per colony) and each queen’s reproductive activity is mediated by queen–queen dominance interactions, even in mature colonies, with highly ranked queens laying more eggs and foraging less frequently outside the nest (Medeiros et al. 1992). In the Brazilian Cerrado savanna, this species is vulnerable to border effects (Christianini and Oliveira 2013), which seem to vary through time (see Salles et al. 2018). Even though *O. chelifer*’s natural history is relatively well studied, little is known about its genetic diversity (but see Larabee et al. 2016, Hoenle et al. 2020).

**Fig. 1.**
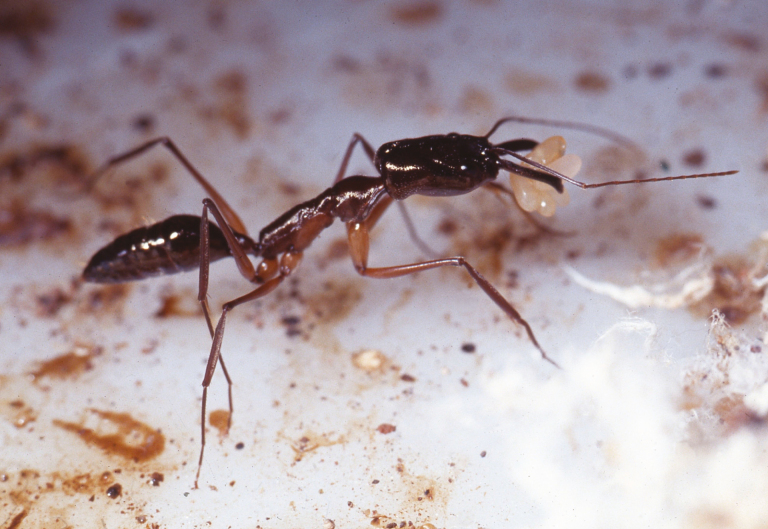
*Odontomachus chelifer* worker carrying eggs (photo by P. S. Oliveira).

Microsatellites—or SSR (simple sequence repeats)—are frequently used as a molecular tool to study genetic diversity in ant colonies. These molecular markers are repetitive sequences (one to six nucleotide repeats), distributed in tandem along the DNA and frequently and randomly distributed across the genome in most eukaryote species (Selkoe and Toonen 2006). Microsatellites are polymorphic, segregate as codominant markers, and are usually selectively neutral (Goldstein and Schlötterer 1999). Several characteristics make them ideal studies on population genetic, genome mapping, and marker-assisted breeding (Sun et al. 2011). Previous works have used microsatellites to identify ant species (Goodisman and Hahn 2005, Ronque et al. 2016), describe their breeding systems (Azevedo-Silva et al. 2020), and analyze dispersion strategies (Pérez-Espona et al. 2012, Soare et al. 2014), colony spatial distribution (Debout et al. 2007), and population genetics patterns (Pamilo et al. 2016). Given that genetic diversity is key for the persistence and adaptation of populations to environmental changes (Seppä 2008), the development of molecular tools to portray such diversity is crucial for elucidating species biology and conservation. Here, we developed and characterized more than 20 microsatellite loci in *O. chelifer*, which will enable future studies on genetic variation and maintenance of *O. chelifer* populations.

## Materials and Methods

### Sampling Site

Colonies of *O. chelifer* were sampled in Fazenda Campininha, a Cerrado savanna reserve located in the city of Mogi-Guaçu (22°18′S e 47°11′W), in the state of São Paulo, southeastern Brazil. Via active searching, we collected 15–20 workers from 18 *O. chelifer* nests. Ant voucher specimens are deposited at the Museu de Zoologia da Universidade Estadual de Campinas (ZUEC, Campinas, Brazil).

### DNA Extraction

DNA extraction was performed using methods described by Saghai-Maroof et al. (1984). In brief, ant individuals are solubilized in 2% CTAB solution, followed by DNA purification through extraction with chloroform/isoamyl alcohol (24:1).

### Microsatellite Identification

Six workers from the same colony of *O. chelifer* were used to build a microsatellite-enriched library, based on protocol previously described in Billotte et al. (1999), using a hybridization-based capture with (CT)_8_ and (GT)_8_ biotin-linked probes, followed by recovery with streptavidin magnetic-coated beads (Promega, Madison, WI). The selected fragments were cloned into the pGEM-T vector (Promega) and transformed into competent *Escherichia coli* (XL1-Blue strain). Recombinant colonies were identified by colorimetric white-blue detection using X-gal. Plasmid DNA was then extracted using an alkaline lysis method and inserts were sequenced on a 3500 Genetic Analyzer sequencer (Applied Biosystems, Foster City, CA). We sequenced 103 clones and the electropherograms were edited using CLC Genomics Workbench v. 4.9 software (CLC Bio, Arhus, Denmark). Vector, adapter, and restriction site sequences were removed using Seqman (DNAStarInc, Madison, WI). Additionally, sequences were compared with entries of the public National Center for Biotechnology Information database using Blastn (Altschul et al. 1990), to eliminate possible contaminant sequences. Microsatellites were identified using the web-based SSRIT software (Temnykh et al. 2001) and primer pairs complementary to their flanking sequences were designed using Primer Select (DNAStarInc) and Primer3Plus (Untergasser et al. 2012). Amplified fragments ranging from 100 to 250 bp in size were selected for further identification of putative alleles. For each sequence, primers were designed according to the following guidelines: 1) 18–22 nucleotides in size, 2) G/C content greater than 35%, 3) T_m_ between 45 and 65°C (maximum of 3°C difference between both primers in the pair), and 4) presence of A or T bases at the 3′ end (to reduce probability of self-pairing). We also avoided designing primers that allow the formation of homo or heterodimers. For automating the genotyping on the 3500 Genetic Analyzer sequencer (Applied Biosystems), an M13 sequence tail (5′-CACGACGTTGTAAAACGAC-3′) was added to the 5′ end of each forward primer (Schuelke 2000). Fluorescent labels 6-FAM, VIC, NED, and PET (Applied Biosystems) were used to increase genotyping efficiency.

### Microsatellite Characterization

Five workers from different nests were used to determine ideal amplification conditions for each developed marker. For all markers, we tested two touchdown PCR protocols (Don et al. 1991), with hybridization temperatures between 52 and 57°C or between 55 and 60°C, together with the following steps: 1) 94°C for 4 min; 2) 10 cycles of 94°C for 45 s, 60°C or 57°C (−0.5ºC/cycle) for 1 min, and 72°C for 1 min and 15 s; 3) 25 cycles of 94°C for 45 s, 50°C for 1 min, and 72°C for 1 min and 15 s; and 4) 72°C for 10 min. The amplification products were evaluated on a 3500 Genetic Analyzer sequencer (Applied Biosystems) following by post-sequencing analysis on Geneious prime software (v. 2019.2; Biomatters Limited, New Zealand).

Microsatellite loci with amplification patterns consistent with expected sizes and clear distinguishable peaks were further characterized for polymorphism content. To this end, we used 30 workers from different nests (Hale et al. 2012), and microsatellite loci were evaluated for occurrence of stuttering and reduced amplification of large fragments using Micro-Checker (Van Oosterhout et al. 2004). Polymorphism content (PIC) (Botstein et al. 1980) and observed and expected heterozygosity for each locus were obtained using the Microsatellites Toolkit supplement in Excel (Park 2008). Rarefied allelic richness was estimated by HP-Rare software (Kalinowski 2005). Additionally, we tested for loci adherence to frequencies expected in the Hardy–Weinberg equilibrium (HWE) using Genepop 4.7 (Rousset 2008). Linkage disequilibrium (LD) between each pair of markers was assessed using FSTAT 2.9.4 (Goudet 1995). For both HWE and LD estimates, the significance value (0.05) was corrected for multiple comparisons. The frequency of null alleles was estimated with the FreeNA software (Chapuis and Estoup 2007).

## Results

The microsatellite enrichment procedure was highly efficient, with 87.85% of the sequenced clones presenting repetitive sequences. Fifty-three clones contained more than one microsatellite sequence, totaling 94 sequences. We were able to design primer pairs for 42 microsatellite loci. Twenty-two loci were successfully amplified using the touchdown PCR protocol with hybridization temperature ranging from 55 to 60°C, and all of them resulted in amplification products consistent with expected sizes (Table 1), without evidence for nonspecific amplification.

**Table 1.** Characteristics of 22 microsatellite loci developed for *Odontomachus chelifer*

Locus	Primer sequences (5′–3′)	Motif	TD (°C)	SR	Ar	HE	HO	PIC	FreqNA	GenBank accession
Och2	F:CACGACGTTGTAAAACGACTGGATGCATGGCTTTAGTCTC R:TTGCGATTAACTCTCATTGGTC	(AG)_4_...(AG)_5_...(AG)_7_...(GA)_39_	60–55	139–239	5.36	0.763	0.455	0.708	0.26735	MT679225
Och3	F:CACGACGTTGTAAAACGACGACAGACTTAATTTTCCTCGTA R:AAATTCGCCGTTGTGAAT	(TC)_29_...(TC)_5_...(CG)_3_...(GC)_4_	60–55	171–231	7.83	0.856	0.9	0.823	0.14942	MT679226
Och6	F:CACGACGTTGTAAAACGACGGCCGTTTCCTGTTCACC R:TCGAAAAAATATTGCCCTCTTC	(CA)_3_...(CA)_21_	60–55	232–292	10.40	0.891	0.5	0.864	0	MT679227
Och8	F:CACGACGTTGTAAAACGACAGTTTTTACAGTGTAGGGCATC R:GAGAAAGCGCCCTTAATCTT	(TC)_3_...(CA)_16_	60–55	136–154	6.68	0.805	0.667	0.766	0.00049	MT679228
Och11	F:CACGACGTTGTAAAACGACTCCTAAATGGTGCGTAATAAAC R:GTATAAAAGGAACGCAGTGAAA	(CA)_9_...(TC)_3_	60–55	190–196	3.65	0.665	0.852	0.584	0	MT679229
Och15	F:CACGACGTTGTAAAACGACAACGCGACGGAAGACGAT R:CGCGAACGTGATATTGGATTAC	(TG)_3_...(AC)_3_	57–52	190–192	1.69	0.078	0	0.074	0	MT679230
Och16	F:CACGACGTTGTAAAACGACGAGAGCGAGAGGGAGTGC R:GAGACATCGATACATCAAAGAG	(GA)_5_...(GA)_5_...(AG)_5_...(AG)_6_...(AG)_10_... (GA)_4_...(GA)_3_...(GA)_5_...(GA)_7_...(GAGA)_3_	60–55	215–265	3.93	0.608	0.167	0.542	0.00100	MT679231
Och27	F:CACGACGTTGTAAAACGACCAATGTGGAGATTACATGGTTA R:GACTAGCAATCCCGAAAACT	(TG)_10_...(TG)_4_	60–55	228–240	3.38	0.678	0.759	0.597	0.13120	MT679232
Och34	F:CACGACGTTGTAAAACGACTAACGATTCATAGACACGCA R:CAGGGAGAATTCCGTTACTG	(AGG)_3_	60–55	136–142	2.38	0.525	0.31	0.399	0.19195	MT679233
Och47	F:CACGACGTTGTAAAACGACTTACCTCCTCGTCACTGAACA R:GAAATCTCGTGCAAAATCTG	(TG)_3_...(AC)_4_...(AC)_19_...(TG)_3_	60–55	238–258	4.61	0.764	0.724	0.709	0.05239	MT679234
Och54	F:CACGACGTTGTAAAACGACGCGATCGAAGCTCGTATTGT R:ACAGTCGGGTCGTAGCACTT	(GGGA)_3_	60–55	163–172	2.98	0.49	0.633	0.432	0.00100	MT679235
Och60	F:CACGACGTTGTAAAACGACAAGGGAAAACGAAAGTGTAATC R:TAACCCGAAACAAGAATACC	(GT)_12_...(TAT)_3_	60–55	182–200	4.55	0.682	0.733	0.613	0.11525	MT679236
Och62	F:CACGACGTTGTAAAACGACTCGCAAGTTAATATCCGTTCAT R:TCTGCTTGGCGAGTGGAC	(CA)_10_	60–55	154–162	3.64	0.6	0.556	0.533	0.00100	MT679237
Och63	F:CACGACGTTGTAAAACGACCGTGGCAGCGCGAAGAAAAA R:GGAAACCCACCCACCGAGACC	(CG)_4_	60–55	175	1	0	0	0	0.03469	MT679238
Och69	F:CACGACGTTGTAAAACGACGCTATGCGTCCCGAACAG R:TCGCCCGACTTGATTGAC	(GA)_3_...(CA)_8_	60–55	216–238	5.28	0.724	0.6	0.673	0.01851	MT679239
Och70	F:CACGACGTTGTAAAACGACGCAATATAAACGCATCTATGTG R:TGTAAACAAACGTAAAAATGG	(AC)_8_	60–55	152–196	3	0.394	0.103	0.344	0.07266	MT679240
Och72	F:CACGACGTTGTAAAACGACCCGTTTATATTGGTATCTTC R:ATATCGCCCATTCCTAAT	(CA)_3_...(AC)_7_	57–52	156–158	2	0.509	0.96	0.375	0.00100	MT679241
Och76	F:CACGACGTTGTAAAACGACGAGAAACGAGTGTGATAAGGTC R:AAAGTCGTTGAAGAATACATCC	(CA)_6_	60–55	173	1	0	0	0	0	MT679242
Och78	F:CACGACGTTGTAAAACGACCGACGGCTAAACCTTCTGAG R:TGACATGGTTTCCTTGAACG	(AT)_4_	60–55	201	1	0	0	0	0	MT679243
Och83	F:CACGACGTTGTAAAACGACGTGCAATATTTTGGATAGAGAA R:TTGTTGTCAGTGGAAAGAATAA	(AT)_3_...(CA)_5_...(CA)_4_...(CACG)_3_	60–55	175–323	3.62	0.572	0.615	0.501	0.21939	MT679244
Och86	F:CACGACGTTGTAAAACGACCGGAATGAAGAATAAAACAGAT R:CTAACAACAGTAACGGCTCAAG	(CT)_3_...(CA)_4_...(CA)_4_...(AC)_3_	60–55	206	1	0	0	0	0.00002	MT679245
Och88	F:CACGACGTTGTAAAACGACCTTTGATTTTTCCAGTAGCACA R:TTACGGTCCTCGAAGTGATTTA	(GC)_8_...(CG)_3_	60–55	135–235	1.81	0.073	0.074	0.071	0.13463	MT679246

For each locus, the table shows primer sequences (F: forward with M13 tail and R: reverse), microsatellite repetitive motif (Motif), amplification temperature range via touchdown PCR (TD), size range after addition of M13 tail (SR), rarified allelic richness (Ar), expected heterozygosity (H_E_), observed heterozygosity (H_O_), polymorphism content (PIC), frequency of null alleles (FreqNA), and GenBank accession number.

Importantly, 18 markers were found to be polymorphic. In these loci, the expected heterozygosity (H_E_) (mean ± SE) was 0.59 ± 0.05, with the highest values found in the *Och6* (0.89), *Och3* (0.86), and *Och8* (0.80) loci. Very low H_E_ values were identified in *Och88* (0.07) and *Och15* (0.08) (Table 1). Rarified allelic richness (mean ± SE) was 4.27 ± 0.53 (Table 1). The mean (±SE) PIC value was 0.53 ± 0.05 (Table 1). We found no evidence of allele stuttering or reduced amplification of fragments for any of the markers. Moreover, the frequency of null alleles ranged from 0 to 0.26735. Regarding adherence to frequencies expected at HWE, we found only seven loci at equilibrium (39%) (Table 1). Finally, it should be noted that all microsatellite loci analyzed exhibited independent segregation.

## Discussion

A microsatellite-enriched library was developed for *O. chelifer*, resulting in 22 markers, 18 of which were found to be polymorphic in the population studied. Even though some markers displayed low polymorphic content, most proved to be highly informative (PIC > 0.5) to access genetic diversity in *O. chelifer* (Table 1). Diversity estimates for our markers were similar to estimates for other ant species in the subfamily Ponerinae. For instance, our results are similar to microsatellite marker allelic richness observed in *Pachycondyla inversa* (5–12 alleles; Trindl et al. 2004), *Hypoponera opacior* (9–21 alleles; Rüger et al. 2005) and *Pachycondyla luteipes* (2–8 alleles; Takahashi et al. 2005), even when rarefied allelic richness is considered in our work.

Despite the availability of microsatellite markers for other Ponerinae or for other ant species (Butler et al. 2014), interchangeability of such markers between species is challenging. This occurs due to their high specificity, which makes cross-amplification limited even at the genus level (Barbará et al. 2007). Thus, developing specific microsatellite loci for each species is a necessity, especially in the context of studies on genetic diversity, reinforcing the importance of the markers developed here for future studies on *O. chelifer* genetic variation.

Eleven microsatellite loci exhibited deviations from HWE, indicating violation of one or more assumptions of the Hardy–Weinberg model, namely presence of selection, migration and/or mutation, finite population size, and non-random mating (Hartl and Clark 2010). Additionally, such deviations may arise from overlapping generations and/or the high relatedness between ant workers. Moreover, our sampling location is a fragmented Cerrado area (Christianini and Oliveira 2013), which may have reduced population size due to habitat loss and/or increased probability of inbreeding (Frankham 2010, Banks et al. 2013). Further investigation is needed to evaluate the effect of habitat fragmentation on genetic variation of *O. chelifer*.

Although the behavior and ecology of *O. chelifer* have already been studied in Brazilian forests and savannas (Oliveira et al. 2017, and references therein), the microsatellite markers described here will stimulate further investigation on colony structure and breeding system in this ant species, including potential effects of habitat fragmentation, a crucial knowledge in the context of conservation of its populations and maintenance of its ecological role.
